# A phase 1 ‘window-of-opportunity’ trial testing evofosfamide (TH-302), a tumour-selective hypoxia-activated cytotoxic prodrug, with preoperative chemoradiotherapy in oesophageal adenocarcinoma patients

**DOI:** 10.1186/s12885-016-2709-z

**Published:** 2016-08-17

**Authors:** Ruben T. H. M. Larue, Lien Van De Voorde, Maaike Berbée, Wouter J. C. van Elmpt, Ludwig J. Dubois, Kranthi M. Panth, Sarah G. J. A. Peeters, Ann Claessens, Wendy M. J. Schreurs, Marius Nap, Fabiënne A. R. M. Warmerdam, Frans L. G. Erdkamp, Meindert N. Sosef, Philippe Lambin

**Affiliations:** 1 Department of Radiation Oncology (MAASTRO), GROW-School for Oncology and Developmental Biology, Maastricht University Medical Centre, Maastricht, The Netherlands; 2 Cancer Research UK & Medical Research Council Oxford Institute for Radiation Oncology, Department of Oncology, University of Oxford, Oxford, UK; 3 Department of Nuclear Medicine, Zuyderland Medical Centre, Sittard-Geleen/Heerlen, The Netherlands; 4 Department of Pathology, Zuyderland Medical Centre, Sittard-Geleen/Heerlen, The Netherlands; 5 Department of Medical Oncology, Zuyderland Medical Centre, Sittard-Geleen/Heerlen, The Netherlands; 6 Department of Surgery, Zuyderland Medical Centre, Sittard-Geleen/Heerlen, The Netherlands; 7 Surgical Collaborative Network Limburg, Limburg, The Netherlands

**Keywords:** Oesophageal cancer, Neoadjuvant chemoradiotherapy, Evofosfamide, Oesophagectomy, Dose limiting toxicity, Hypoxia imaging, Window-of-opportunity trial

## Abstract

**Background:**

Neo-adjuvant chemoradiotherapy followed by surgery is the standard treatment with curative intent for oesophageal cancer patients, with 5-year overall survival rates up to 50 %. However, patients’ quality of life is severely compromised by oesophagectomy, and eventually many patients die due to metastatic disease.

Most solid tumours, including oesophageal cancer, contain hypoxic regions that are more resistant to chemoradiotherapy. The hypoxia-activated prodrug evofosfamide works as a DNA-alkylating agent under these hypoxic conditions, which directly kills hypoxic cancer cells and potentially minimizes resistance to conventional therapy. This drug has shown promising results in several clinical studies when combined with chemotherapy. Therefore, in this phase I study we investigate the safety of evofosfamide added to the chemoradiotherapy treatment of oesophageal cancer.

**Methods/Design:**

A phase I, non-randomized, single-centre, open-label, 3 + 3 trial with repeated hypoxia PET imaging, will test the safety of evofosfamide in combination with neo-adjuvant chemoradiotherapy in potentially resectable oesophageal adenocarcinoma patients. Investigated dose levels range from 120 mg/m2 to 340 mg/m2. Evofosfamide will be administered one week before the start of chemoradiotherapy (CROSS-regimen) and repeated weekly up to a total of six doses. PET/CT acquisitions with hypoxia tracer ^18^F-HX4 will be made before and after the first administration of evofosfamide, allowing early assessment of changes in hypoxia, accompanied with blood sampling to measure hypoxia blood biomarkers. Oesophagectomy will be performed according to standard clinical practice.

Higher grade and uncommon non-haematological, haematological, and post-operative toxicities are the primary endpoints according to the CTCAEv4.0 and Clavien-Dindo classifications. Secondary endpoints are reduction in hypoxic fraction based on ^18^F-HX4 imaging, pathological complete response, histopathological negative circumferential resection margin (R0) rate, local and distant recurrence rate, and progression free and overall survival.

**Discussion:**

This is the first clinical trial testing evofosfamide in combination with chemoradiotherapy. The primary objective is to determine the dose limiting toxicity of this combined treatment and herewith to define the maximum tolerated dose and recommended phase 2 dose for future clinical studies. The addition of non-invasive repeated hypoxia imaging (‘window-of-opportunity’) enables us to identify the biologically effective dose. We believe this approach could also be used for other hypoxia targeted drugs.

**Trial registration:**

ClinicalTrials.gov Identifier: NCT02598687.

## Background

The incidence of oesophageal cancer in developed Western countries has risen in recent decades [1]. Adenocarcinoma is now more prevalent than squamous cell carcinoma, with most tumours located in the distal oesophagus. A Western lifestyle is a risk factor and the disease is associated with obesity and symptomatic gastro-oesophageal reflux [2]. The standard treatment with curative intent for T2 or higher stage tumours consists of neoadjuvant chemoradiotherapy (nCRT) followed by surgery, [3] as confirmed by the Dutch ChemoRadiotherapy for Oesophageal cancer followed by Surgery Study (CROSS) [4]. In this study, significantly better 5-year overall survival (OS) rates were observed for patients treated with nCRT followed by surgery (47 %; 95 % CI 39–54) when compared to surgery alone (33 %; 95 % CI 26–40), with greater benefits for squamous cell carcinoma (61 % vs. 30 %) than for adenocarcinoma (43 % vs. 33 %) [5]. However, little progress has been made in long-term survival (median OS ~49 months) and the patients’ quality of life is still severely compromised by the impact of oesophagectomy. Therefore, there is an urgent need for new innovative treatment strategies.

The role of the tumour microenvironment in cancer progression, and especially the difference between this microenvironment and surrounding normal tissue, is a subject of increasing investigational interest with a specific focus on hypoxia. Hypoxic tumour cells promote a more aggressive phenotype, are associated with increased metastatic potential, and are known to be more resistant to standard chemoradiotherapy [6–14]. Recently, even micro-metastases have been shown to exhibit hypoxia [15]. Up to 5–10 % of the oesophageal cancer patients suffer from progressive disease with metastases shortly after completion of neoadjuvant chemoradiotherapy [16] and the majority of patients eventually die because of metastatic disease. Therefore, hypoxia is an attractive target for newly developed drugs to increase the therapeutic effect of conventional oesophageal cancer treatment modalities.

Evofosfamide (TH-302) is a hypoxia-activated prodrug only activated under low levels of oxygen (hypoxia) [17–23]. Evofosfamide exploits the activation of a nitroimidazole prodrug by a process that involves the reduction of one electron, mediated by ubiquitous cellular reductases as the NADPH cytochrome P450 reductase to generate a radical anion prodrug. In the presence of oxygen (normoxia) the radical anion prodrug reacts rapidly with oxygen to produce superoxide and re-generate the original prodrug. Under the low-oxygen conditions of the hypoxic zones in tumours, however, the radical anion form of the prodrug has a longer half-life and can either fragment directly, or undergo further reductions, releasing the active drug bromo-isophosphoramide mustard that acts as a DNA cross-linker.

**Fig. 1:**
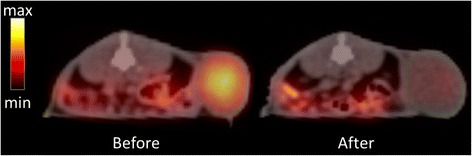
Evofosfamide decreases the hypoxic fraction in a Rhabdomyosarcoma rat model. PET-scans with hypoxia tracer ^18^F-HX4 were made before (day 0) and after (day 4) administering evofosfamide for four consecutive days at a dose of 25 mg/kg

Recently, our group reported the radio-sensitizing effect of evofosfamide in a preclinical setting using syngeneic rat R1 rhabdomyosarcoma and human H460 NSCLC (non-small cell lung cancer) xenograft tumour models. Evofosfamide treatment significantly reduced the hypoxic fraction, by more than 80 % compared to the control tumours in both tumour models. This was visualized at either micro-regional level or on PET images with the hypoxia tracer ^18^F-HX4 (Fig. 1). Treatment with evofosfamide alone caused a significant delay in tumour growth while, when combined with radiotherapy (8 Gy), the growth delay was further enhanced. In addition, hypoxic fractions determined by pre-treatment ^18^F-HX4 scans were predictive for the response associated with evofosfamide treatment. Therefore, a pre-treatment ^18^F-HX4 scan may be beneficial for selection of patients for evofosfamide treatment and a post treatment ^18^F-HX4 scan enables to monitor treatment efficacy [20].

Evofosfamide has already been clinically investigated both as monotherapy and in combination with chemotherapy or other targeted cancer drugs in over 1,500 patients, and is currently under investigation in more than fifteen clinical trials registered at clinicaltrials.gov. Investigated tumours include soft tissue sarcoma, pancreatic cancer, non-small cell lung cancer, melanoma, and haematological malignancies. In general, the drug is well tolerated with main toxicities being higher-grade skin and mucosal toxicities, in particular in doses above 240 mg/m^2^ [24, 25]. In patients with advanced pancreatic cancer or soft-tissue sarcoma the combination of chemotherapy with evofosfamide achieved favourable outcomes, [26, 27] leading to two phase III clinical trials (NCT01746979 and NCT01440088). Despite the promising pre-clinical results demonstrating the potential added value of evofosfamide in combination with radiotherapy [20], to date no clinical studies have been performed to confirm this.

The primary objective of this 3 + 3 dose escalation phase I ‘window-of-opportunity’ trial is to investigate the safety of evofosfamide in combination with the standard CROSS regimen in patients with distal oesophageal and oesophago-gastric junction adenocarcinoma, to determine the dose-limiting toxicities (DLTs) of the combined regimen and consequently to find the maximum tolerated dose (MTD) and recommended phase II dose (RP2D). As ^18^F-HX4 has shown to be a hypoxia PET-tracer [28–35] with good repeatability in oesophageal cancer [36], two ^18^F-HX4 PET-scans will be performed to characterize tumour hypoxia at baseline and visualize the potential change in hypoxia after the first administration of evofosfamide [9, 37].

## Methods/Design

### Study design

This is a phase I, non-randomized, single-centre, open-label, 3 + 3, ‘window-of-opportunity’ trial combining preoperative evofosfamide with the CROSS regimen (NCT02598687).

**Fig. 2:**
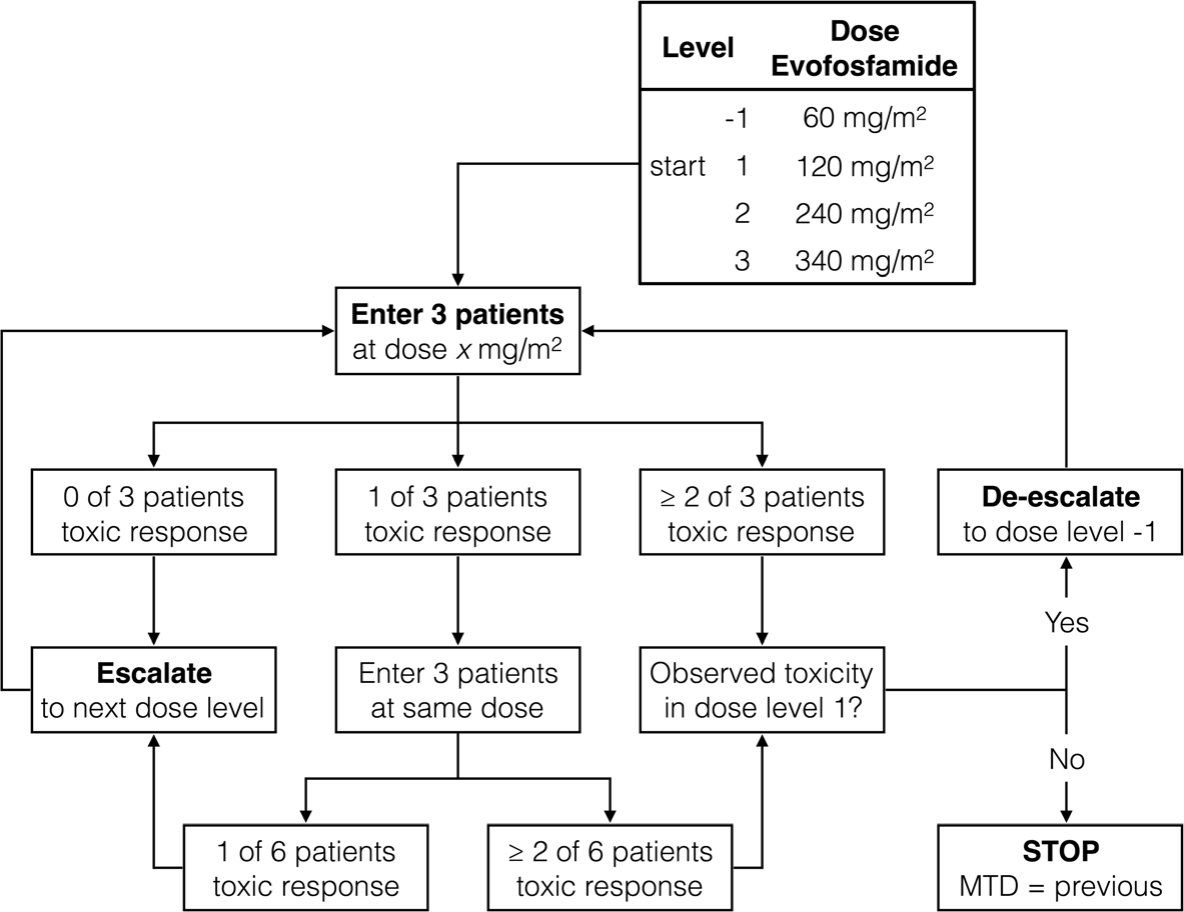
Flowcharts summarizing the 3 + 3 dose escalating study design. In the first cohort of patients evofosfamide will be administered at a dose of 120 mg/m2. Depending on the observed toxicity, we will escalate to dose level 2, or de-escalate to dose level -1. In further dose levels we can only escalate to the next dose (up to 340 mg/m2) or stop due to dose limiting toxicity

In this traditional 3 + 3 dose-escalation design [38, 39], a cohort of three patients will enter a given dose level, and if no dose limiting toxicity (DLT) is observed 30 days after surgery, the trial will proceed to the next dose level. If a DLT occurs in 1 of 3 patients at a given dose level, 3 additional patients will be added to the same dose level cohort. If the occurrence of DLT remains limited to 1 out of 6 patients, the trial will proceed to the next dose level. If a DLT occurs in 2 or more patients at a certain dose level, dose escalation will be stopped. The previous dose level is then considered the maximum tolerated dose (MTD) and, therefore, the recommended dose for a phase II study. This is also summarized in Fig. 2. In this study three dose levels will be tested, which means that a maximum of nine to eighteen patients will be included.

### In- and exclusion criteria

Prior to treatment, patients are discussed at the centralized multidisciplinary tumour board consisting of, inter alia, a surgeon, medical oncologist, nuclear medicine physician and radiation oncologist. Potentially curable patients with histologically proven stage IB-IIIC T2-4 distal oesophageal or oesophago-gastric junction adenocarcinoma are eligible to participate in this study. The minimum age is 18 years and the patients need to be fit for chemoradiotherapy with a normal baseline electrocardiogram (ECG) and a performance status of 0–2 according to the World Health Organisation (WHO) classification.

The most important exclusion criteria are: a history of thoracic radiotherapy, recent severe cardiac or pulmonary disease, pregnancy, and/or viral infection.

### Study treatment

**Fig. 3:**
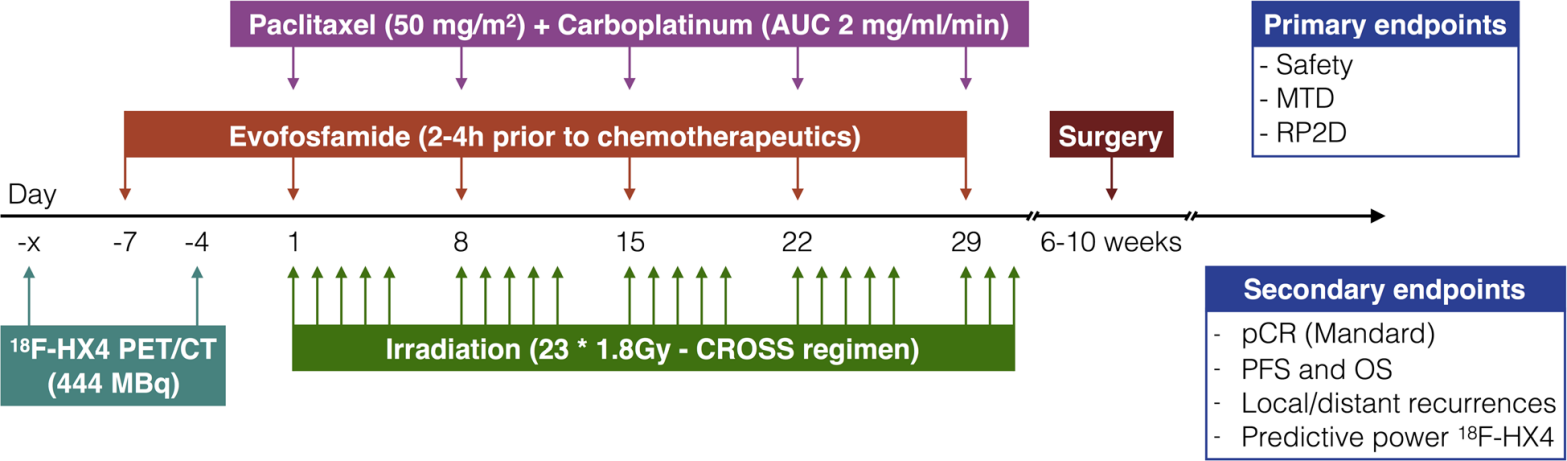
Study treatment schedule. Prior to the start of the standard CROSS treatment, patients will receive additional ^18^F-HX4 scans before and after the first dose of evofosfamide

All patients will receive nCRT according to the CROSS regimen (Carboplatin AUC = 2 mg/ml/min, Paclitaxel 50 mg/m^2^, concurrent radiotherapy 41.4 Gy/23 fractions) [4]. One starting dose of evofosfamide will be administered one week prior to the start of the nCRT, after which five additional administrations will be given weekly on the same day as Carboplatin and Paclitaxel, but 2–4 h prior to the chemotherapeutics (Fig. 3). The drugs will be administered via a peripherally inserted central catheter (PICC) and an ECG will be made before and after administration of the first two fractions of evofosfamide. Investigated dose levels range from 120 mg/m^2^ to 340 mg/m^2^ evofosfamide, with the possibility to de-escalate to a dose level of 60 mg/m^2^ (Fig. 2). Due to the activation of evofosfamide under all hypoxic conditions, the patients will receive extra skin-care, e.g. by using cold packs during administration, to reduce hypoxia and prevent the possible occurrence of any severe skin-toxicities.

Surgical resection will be attempted six to ten weeks after completion of the nCRT, depending on the patient’s characteristics and the lack of evidence for metastatic disease on a re-evaluation FDG-PET/CT-scan made before the planned surgery. Depending on tumour location and general comorbidity, either a minimally invasive transhiatal approach, including a one-field and low mediastinal lymph node dissection, or a transthoracic approach with a two-field lymph node dissection will be performed. The pathological tumour response of the resected specimen will be evaluated using the standardized pathology protocol, reporting, amongst others, the tumour regression grade according to the Mandard scoring [40], and the status of the resected lymph nodes and resection margins.

### Imaging

Standard non-invasive diagnostic modalities include a (whole-body) FDG-PET/CT scan and/or endoscopic ultrasound (EUS) with biopsy. Patients that are included in this study will receive two additional PET/CT-scans with the hypoxia tracer ^18^F-HX4: the first scan at baseline and the second scan three days after the first administration of evofosfamide (Fig. 3). ^18^F-HX4 will be administered via a bolus intravenous injection 444 MBq (12 mCi), and the PET/CT-scan will be acquired 4 h post injection. The detailed acquisition protocol was previously described by Zegers et al. [33]. Based on the available diagnostic information, only a single bed position centred around the primary tumour site will be imaged and a slice-thickness of 3 mm will be used for the CT-reconstruction [28, 29, 33, 34]. On both days a blood sample (5–7 ml) will be drawn before administration of ^18^F-HX4 to analyse the concentration of hypoxia related blood biomarkers carbonic anhydrase IX (CAIX) and osteopontin (OPN) [41].

### Study parameters and endpoints

The main study endpoint is to determine the DLT and define the MTD and RP2D. DLT is defined as:

- Uncommon grade 3 or higher non-haematological toxicity according to the Common Terminology Criteria for Adverse Events (CTCAE) version 4.0. Grade III esophagitis in 50 % of the patients is accepted.
- Grade 4 or higher haematological toxicity according to CTCAE version 4.0.
- Grade 4 or higher postoperative toxicity within 30 days post-surgery according to the Clavien-Dindo classification [42]. For anastomotic leakage and cardiorespiratory complication we accept a rate of 50 % and 40 % respectively.
- Any grade 2 or higher non-haematological toxicity that does not resolve to grade 0 or 1 toxicity by the start of the next cycle, which is considered a DLT according to the judgement of the investigator or sponsor.
- Inability to begin the next cycle of treatment within two weeks of the last dose due to unresolved toxicity.

Secondary endpoints include investigating the change in hypoxia based on ^18^F-HX4 imaging and blood biomarkers to explore what would be the biologically effective dose, and anti-tumour activity measured by the rate of pathological complete response (pCR), histopathological negative circumferential resection margin (CRM) rate, local and distance recurrence rate, and progression free and overall survival.

### Post-treatment

End of treatment is defined as the date of the last radiotherapy fraction in case of treatment completion according to protocol. When a patient drops-out before the end of treatment for any reasons other than DLT, he or she will be replaced by an additional patient.

If there is a complete remission on re-evaluation FDG-PET/CT-scan after chemoradiotherapy, a patient can discuss the possibility for a wait-and-see strategy off protocol [43]. The decision to proceed to the next dose level of evofosfamide will be made when the minimum post-surgery or post-chemoradiotherapy (if no further surgery) follow-up of each patient in a particular dose level is 30 days.

Patients are examined weekly during the treatment. Follow-up starts directly after the end of treatment and adverse events will be assessed at 1 and 4 weeks after nCRT, right before surgery and one month after surgery. Thereafter, follow-up visits will be planned every three months in the first year after nCRT, twice in the second year, and then yearly until a minimum follow-up time of five years.

## Discussion

### Known and potential risks and benefits

The primary dose limiting toxicities of evofosfamide from clinical studies have indicated more haematological toxicity than in monotherapeutic chemotherapy. Skin and mucosal toxicities are common above doses of 240 mg/m^2^ [24, 25]. The mucosal toxicities increase with dose but are still treatable with conservative approaches. The percentage of grade 3 esophagitis is expected to be higher in our proposed study design than with the standard CROSS treatment, but can be managed adequately (feeding tube, parenteral nutrition).

Evofosfamide has not been tested in combination with carboplatin, paclitaxel and radiotherapy before, so no pharmacological interactions between these drugs are known. A potential risk is that all three drugs can cause mild haematological toxicity (reversible leukopenia, neutropenia and/or lymphopenia) in some patients, but it is not known if this effect will be amplified by combining the drugs. Therefore we start with low dose levels of evofosfamide in comparison to the maximum tolerated doses in previous clinical studies. As an extra safety measure, patients in the first cohort will only be included when the previously included patient has finished chemoradiotherapy.

We are aware that with our trial design, combining evofosfamide with trimodality treatment, it will be difficult to determine the exact cause of potential adverse events. An alternative design would be to only include oesophageal cancer patients that receive either chemotherapy (carboplatin and/or paclitaxel) or radiotherapy. However, this strategy currently is only applied in very rare palliative cases. Moreover, the dose levels of chemotherapy and radiotherapy in a palliative setting are different than in a curative setting. Potentially curable patients will always receive trimodality therapy and withholding one of the treatment modalities to test the safety in combination with evofosfamide in potentially curable patients would, obviously, be very unethical, especially since the effectiveness of evofosfamide in oesophageal cancer is not proven yet. Hence, we believe that it is essential to test evofosfamide in the setting as proposed in this trial.

The benefit of this ‘window-of-opportunity’ trial is that the clinical activity of evofosfamide in patients with oesophageal cancer can be studied without being compromised by previous or interfering treatments [37]. Another benefit is that the combination therapy might overcome resistance to conventional treatment with chemoradiotherapy and creates a supra-additive effect with increased tumour response. Patients with a complete pathological response after neo-adjuvant treatment eventually could opt for a wait-and-see strategy to omit or postpone surgery.

### Explorative image analysis

Hypoxia PET-tracer ^18^F-HX4 has been extensively used at our institute in both pre-clinical and clinical studies. It is shown that the tracer is not associated with any toxicity [31], has a stable uptake pattern [33], provides complementary information to metabolic FDG imaging [34], and has a good spatial stability in lung, head and neck [29], and oesophageal cancer [36]. The design of this trial enables us to study the hypoxic response based on imaging biomarker ^18^F-HX4 and blood biomarkers CAIX and OPN. This early response assessment will give us insight into the anti-tumour activity of evofosfamide, and can be used to define the optimal dose for future clinical research.

Calais et al. showed previously that high FDG-uptake regions at baseline identify tumour sub-volumes that are at a greater risk of recurrence [44]. Therefore explorative image analysis will be performed to visualize the spatial correlation between the baseline FDG-uptake and ^18^F-HX4 uptake first, and later investigate if the high uptake regions correlate with the patterns of residual disease. Also the correlation between ^18^F-HX4-imaging and hypoxia blood biomarkers CAIX and OPN will be evaluated.

Predictive models of outcome (e.g. pathological response, survival) will be developed based on a so-called Radiomics analysis. Radiomics is the extraction of a large number of quantitative intensity, shape and textural features from both CT and PET images [45]. It was shown that Radiomics features have prognostic value in both lung and head and neck cancer [46–48]. The additional value of Radiomics features in response prediction of oesophageal cancer patients is currently under investigation [49–51].

Another phase II clinical study currently investigates the effect of tumour hypoxia on the response to standard chemoradiation, by visualizing hypoxia with ^18^F-HX4-imaging before treatment and two weeks after the start of treatment (NCT02584400). Since the HX4-scanning settings are identical, the imaging data of both studies provide complementary information about the behaviour and influence of tumour hypoxia in oesophageal cancer treatment. All of this together may be of additional value to better stratify patients in the future [52], by identifying patients who would benefit from hypoxia-selective treatment, such as evofosfamide, already in an early stage.

## Abbreviations

^18^F-HX4: [^18^F]-fluortanidazole
AUC: Target area under the concentration-time curve in mg/ml/min
CAIX: Carbonic anhydrase IX
CRM: Circumferential resection margin
CROSS: ChemoRadiotherapy for Oesophageal cancer followed by Surgery Study
CT: Computed tomography
CTCAE: Common toxicity criteria for adverse events
DLT: Dose-limiting toxicity
ECG: Electrocardiogram
EUS: Endoscopic ultrasound
FDG: [^18^F]-fluordeoxyglucose
MTD: Maximum tolerated dose
nCRT: Neo-adjuvant chemoradiotherapy
OPN: Osteopontin
OS: Overall survival
pCR: Pathological complete remission
PET: Positron emission tomography
PICC: Peripherally inserted central catheter
RP2D: Recommended phase 2 dose
TH-302: Former abbreviation for evofosfamide
WHO: World Health Organisation
